# Clusterin Deficiency Exacerbates Hyperoxia-Induced Acute Lung Injury

**DOI:** 10.3390/cells10040944

**Published:** 2021-04-19

**Authors:** Jung Yeon Hong, Mi Na Kim, Eun Gyul Kim, Jae Woo Lee, Hye Rin Kim, Soo Yeon Kim, Soon Min Lee, Yoon Hee Kim, Kyung Won Kim, Myung Hyun Sohn

**Affiliations:** Department of Pediatrics, Severance Hospital, Institute of Allergy, Brain Korea 21 Project for Medical Science, Yonsei University College of Medicine,134 Sinchon-Dong, Seoul 03722, Korea; hjy2001@yuhs.ac (J.Y.H.); SKDIAALSK@yuhs.ac (M.N.K.); DKALRYA@yuhs.ac (E.G.K.); JWLEE1213@yuhs.ac (J.W.L.); HYERIN0216@yuhs.ac (H.R.K.); SOPHI1@yuhs.ac (S.Y.K.); smlee@yuhs.ac (S.M.L.); YHKIM@yuhs.ac (Y.H.K.); KWKIM@yuhs.ac (K.W.K.)

**Keywords:** clusterin, acute lung injury, hyperoxia, apoptosis, inflammation

## Abstract

Exposure to high oxygen concentrations leads to generation of excessive reactive oxygen species, causing cellular injury and multiple organ dysfunctions and is associated with a high mortality rate. Clusterin (CLU) is a heterodimeric glycoprotein that mediates several intracellular signaling pathways, including cell death and inflammation. However, the role of CLU in the pathogenesis of hyperoxic acute lung injury (HALI) is unknown. Wild-type (WT) and CLU-deficient mice and cultured human airway epithelial cells were used. Changes in cell death- and inflammation-related molecules with or without hyperoxia exposure in cells and animals were determined. Hyperoxia induced an increase in CLU expression in mouse lungs and human airway epithelial cells. Mice lacking CLU had increased HALI and mortality rate compared with WT mice. In vitro, CLU-disrupted cells showed enhanced release of cytochrome c, Bax translocation, cell death and inflammatory cytokine expression. However, treatment with recombinant CLU attenuated hyperoxia-induced apoptosis. Moreover, the Kyoto Encyclopedia of Genes and Genomes and Gene Ontology analyses revealed metabolic pathways, hematopoietic cell lineage, response to stress and localization and regulation of immune system that were differentially regulated between WT and CLU^−/−^ mice. These results demonstrate that prolonged hyperoxia-induced lung injury is associated with CLU expression and that CLU replenishment may alleviate hyperoxia-induced cell death.

## 1. Introduction

Oxygen supplementation is an important therapeutic strategy for respiratory failure in patients with severe pneumonia, acute lung injury and in premature infants [1]. However, prolonged administration of a high concentration of oxygen can exacerbate hyperoxic acute lung injury (HALI) [2]. HALI is mainly characterized by excessive pulmonary inflammation, destruction of capillary endothelial cells with capillary leak, thickening of the alveolar-capillary barrier and promotion of cell injury and death [3]. This damage induces a cascade of pro-inflammatory cytokines and pro-apoptotic signals, such as interleukin (IL)-1β, IL-6, Bax, cytochrome C, reactive oxygen species (ROS) and caspases [2,4]. Among the many features of HALI, airway epithelial cell damage and apoptosis are considered to be important in the pathogenesis of HALI [4,5] because the condition of airway epithelial cells directly affects the degree of lung injury [6].

Clusterin (CLU) is a heterodimeric glycoprotein that is widely distributed in many tissues and fluids and regulated by a variety of environmental stresses. CLU has been implicated in a number of physiologic and pathologic processes, such as immune modulation, cell death, cell-cycle regulation, DNA repair, tissue differentiation and remodeling, lipid transportation and cancer progression [7,8,9]. Accordingly, CLU has been reported to be overexpressed following increased oxidative stress in several cell lines, including airway epithelial cells [10], osteosarcoma cells [11] and cardiomyocytes [12]. Recently, studies on CLU-knockout mice have found that CLU deficiency accelerates lung damage in response to bleomycin [13] and in mice with allergic airway inflammation [9]. Further, CLU-deficient mice exhibited greater severity of lung inflammation, as well as the recruitment of eosinophils, dendritic cells and monocytes to the lungs after house dust mite inhalation [9]. Moreover, it is involved in the inhibition of apoptotic signals after oxidative injury through molecular interactions mediated by phosphorylated PI3K/AKT, NFκB and MAP kinases [12,14]. Reportedly, CLU expression is markedly higher in biopsy specimens obtained from a current smoker and an ex-smoker than in the control samples. Experimentally, exposure of human lung fibroblasts to cigarette smoke results in the release of CLU and its accumulation to protect lung fibroblasts against cigarette smoke-induced oxidative stress [15]. Although CLU is associated with several disorders, its role in hyperoxia-induced acute lung injury has not yet been clarified. Hence, we hypothesized that CLU exerts protective effects on hyperoxia-induced lung injury and cell death by regulating mitochondrial disruption and inflammatory mediator release. Therefore, this study aimed to evaluate the effect of hyperoxia on CLU expression and the effect of CLU on hyperoxia–induced lung damage and cell death in both mice and human airway epithelial cells. Using CLU null mutant mice (CLU^−/−^ mice), we showed CLU to play a protective role during acute lung injury. Furthermore, CLU exhibited significant apoptosis-inhibitory effects and decreased the release of apoptotic factors, thereby regulating mitochondrial alteration [16].

## 2. Materials and Methods

### 2.1. Animals

All experiments used 8–10-week-old, male and age-matched mice, housed under specific pathogen-free conditions. C57BL/6 mice (Orient Bio, Daejeon, Korea) and CLU^−/−^ mice with the same genetic background (Jackson Laboratories, Bar Harbor, ME, USA.) were used. Throughout the experiment, mice were allowed access to food and water ad libitum and maintained on a 12 h dark–light cycle. Animal experiments were performed in compliance with the guidelines of the Korea Research Institute of Bioscience and Biotechnology and according to the Laboratory Animals Welfare Act, the Guide for the Care and Use of Laboratory Animals and the Guidelines and Policies for Rodent Experiment provided by the Institutional Animal Care and Use Committee and were approved by the Institutional Animal Care and Use Committee of the Yonsei University Health System. (IACUC no.2018-0010).

### 2.2. Oxygen Exposure

The animals were divided into four groups: (1) wild-type room air control (WT/RA), (2) CLU^−/−^ room air control (CLU^−/−^/RA), (3) wild-type hyperoxia group (WT/HO) and (4) CLU^−/−^ hyperoxia group (CLU^−/−^/HO). For inducing hyperoxia-induced acute lung injury, mice were placed in cages in a Plexiglas chamber (57 × 42 × 27 cm^3^, JEUNGDO Bio & Plant Co., Ltd., Seoul, Korea), which was continuously supplied with O_2_ at 5 L/min for 72 h, as reported previously [1]. To maintain O_2_ saturation at >95% and prevent CO_2_ accumulation, the level of O_2_ inside the chamber was constantly monitored using an oxygen analyzer (MaxO2^+^A, MAXTEC) (Salt Lake City, UT, USA.). Age- and sex-matched WT and CLU^−/−^ mice were kept under normoxia in similar conditions to serve as room air controls. After 72 h of O_2_ exposure, all the mice were sacrificed and bronchoalveolar lavage fluid (BALF) and lung tissues were harvested.

### 2.3. RNA Isolation and Quantitative Real-Time PCR

RNA was extracted using TRIzol reagent (Thermo Scientific, Waltham, MA, USA.) from lungs perfused with PBS or cells and cDNA was synthesized as previously described [17,18]. Furthermore, the expression of mRNAs was assessed by real-time PCR using Power SYBR™ Green PCR Master Mix (Applied Biosystems, Foster City, CA, USA.) with a StepOnePlus Real-Time PCR System (Applied Biosystems). Thermal cycling was initiated with an initial denaturation step of 5 min at 95 °C, followed by 40 cycles of 95 °C for 30 s and 60 °C for 30 s. The sequences of the primers used for PCR were as follows: mCLU (F: 5′-AGCAGGAGGTCTCTGACAATG-3′; R: 5′-GGCTTCCTCTAA ACTGTTGAGC-3′), mFAS (F: 5′-ATGCACACTCTGCGATGAAG-3′; R: 5′-TTCAGGGTCATCCTGTCTCC-3′), mIL-6 (F: 5′-CTGCAAGAGACTTCCATCCAG-3′; R: 5′-AGTGGTATAGACAGGTCTGTTGG-3′), mTNF-α (F: 5′-GGCAGGTCTACTTTGGAGTCATTGC-3′; R: 5′-ACATTCGAGGCTCCAGTGAATTCGG-3′), mHPRT (F: 5′-GGAATTTGAATCACGTTTGT-3′; R: 5′- TCAACAG GACTCCTCGTATT-3′), hCLU (F: 5′-ATGATGAAGACTCTGCTGCTG-3′; R: 5′-TTCCTGGAGCTCATTGTCTG-3′), hIL-1β (F: 5′-CTGTCCTGCGTGTTGAAAGA-3′; R: 5′-TTCT GCTTGAGAGGTGCTGA-3′), hTNF-α (F: 5′-AACCTCCTCTCTGCCATCAA-3′; R: 5′-CCAAAGTAGACCTGCCCAGA-3′) and h18srRNA (F: 5′-GTAACCCGTTGAACCCCATT-3′; R: 5′-CCATCCAATCGGTAGTAGCG-3′). The comparative cycle threshold method was used to analyze the relative fold changes in gene expression.

### 2.4. Statistical Analysis

Data are expressed as mean ± standard deviation (SD). Statistical analyses for human data were performed using R (version 3.3.3; R Foundation for Statistical Computing, Vienna, Austria). Categorical data are presented as count and percentages. Continuous data are reported as the mean (±standard deviations) or median (interquartile range), as appropriate. Comparisons of two groups were conducted by Student’s *t*-test or Mann–Whitney *U* test for continuous variables and chi-square test or Fisher’s exact test for categorical variables. Statistical significance was defined as a *p*-value less than 0.05.

## 3. Results

### 3.1. Hyperoxia-Induced Acute Lung Injury Is Associated with Elevated Levels of CLU

**Figure 1:**
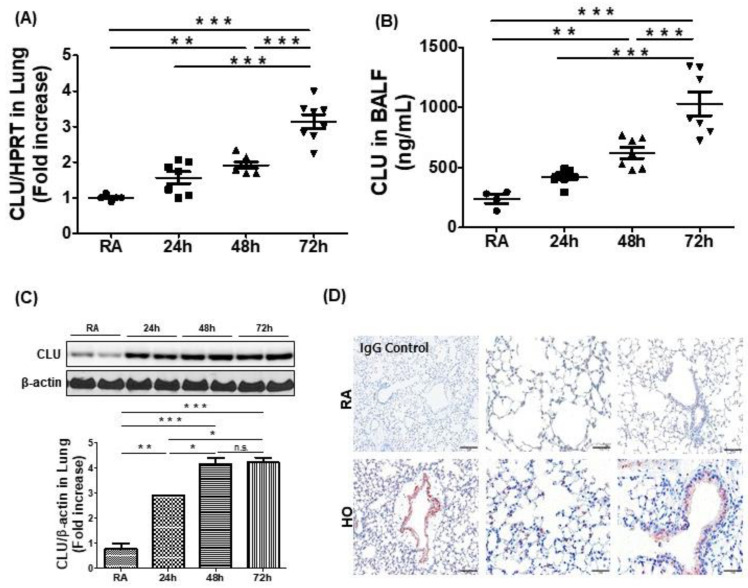
Hyperoxia induces clusterin (CLU) production in mouse lung. Wild-type (WT) mice were exposed to room air (RA) or >95% O_2_ (HO) for up to 72 h. (**A**) Time-course of CLU expression as measured by real-time PCR in the lung tissues of mice exposed to either HO or RA control. (**B**) The level of CLU in bronchoalveolar lavage fluid was assessed using ELISA. (**C**) Pulmonary CLU protein expression was assessed via Western blotting. (**D**) CLU immunohistochemistry in lung sections exposed to HO or RA control. The images are representative of a minimum of 4 mice per group. Each symbol in A and B represents the value for every mouse. Scale bars in (**D**); 100 μm; n.s. = not significant; * *p* < 0.05, ** *p* < 0.01, *** *p* < 0.001.

To determine the role of CLU in hyperoxia, we evaluated the expression of airway CLU in the BALF and lungs of C57BL/6 mice exposed to room air and hyperoxia (≥95% O_2_; HO) for up to 72 h. Total RNA was isolated from the lungs of hyperoxia-exposed mice or room air controls. Real-time PCR revealed that CLU was significantly elevated at 48 h and 72 h as compared with levels in room air controls (Figure 1A). Furthermore, the levels of CLU protein were significantly increased in both BALF and lungs of hyperoxia-exposed mice compared with levels in room air controls (Figure 1B,C). We performed immunolocalization for CLU in hyperoxia-exposed lung tissue. As shown in Figure 1D, CLU was predominantly expressed on airway epithelial cells and vascular endothelial cells in the lungs of hyperoxia-exposed mice.

### 3.2. CLU Protects Against Hyperoxia-Induced Lung Injury and Apoptosis in Mice

**Figure 2:**
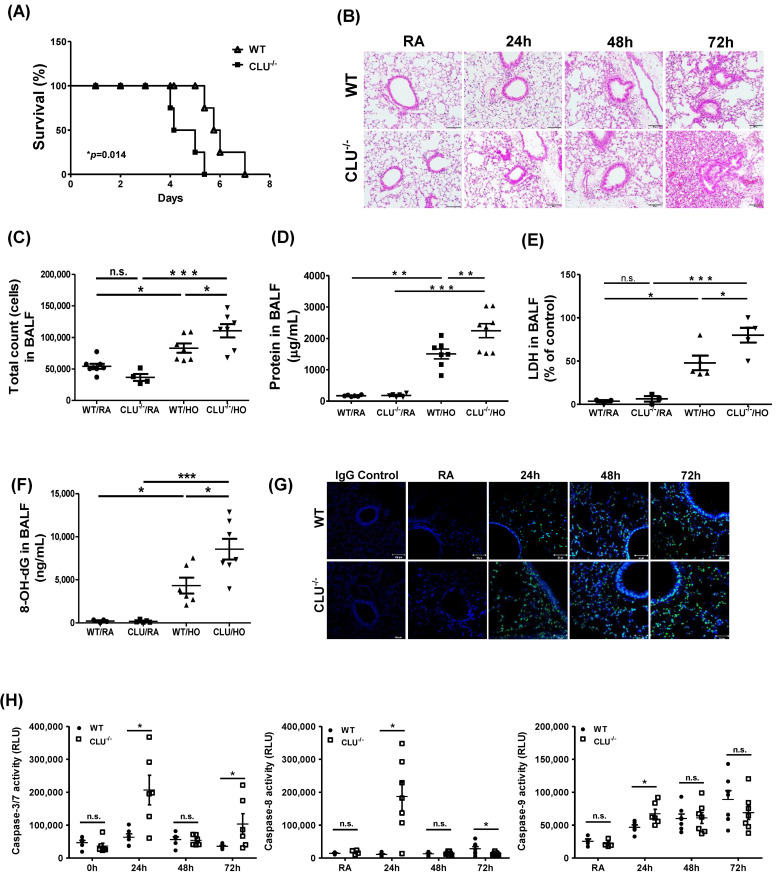

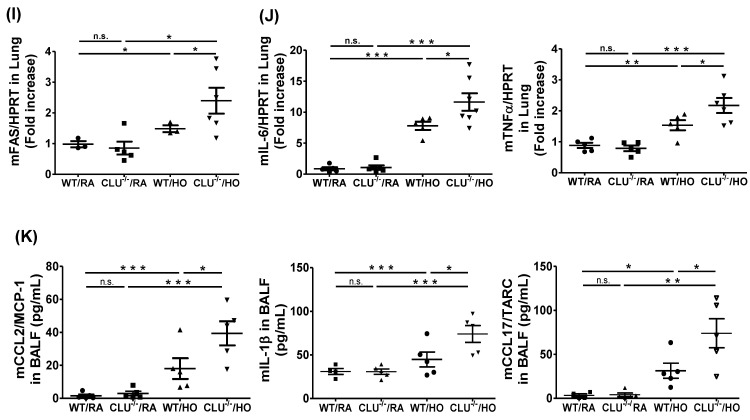
Clusterin (CLU) deficiency exacerbates hyperoxia-induced lung injury. Wild-type (WT) and CLU-deficient (CLU^−/−^) mice were exposed to >95% O_2_ and (**A**) survival, (**B**) lung tissue injury (assessed by light microscopy, H&E staining), (**C**) BALF total cell recovery and (**D**) BALF protein (**E**) LDH and (**F**) 8-OH-dG levels were assessed. (**G**) TUNEL staining of lung tissue sections. Sections were stained with TUNEL (green) and DAPI (blue). (**H**) Caspase-3/7, -8 and -9 activities were measured in the lung lysates. The mRNA levels of (**I**) Fas and (**J**) IL-6 and TNF-α were detected by real-time PCR. (**K**) CCL2, IL-1β and CCL17 expression levels were determined in BALF. The data represent assessments in a minimum of *n* = 5 mice. Each symbol in dot plot graph represents the value for every mouse. Scale bars in (**B**): 100 μm; Scale bars in (**G**): 50 μm; n.s. = not significant; * *p* < 0.05, ** *p* < 0.01, *** *p* < 0.001.

To elucidate the effect of CLU in the development of HALI, CLU^−/−^ mice were exposed to hyperoxia for 72 h. Compared with WT mice, CLU^−/−^ mice showed increased mortality under hyperoxic conditions (*p* = 0.014) (Figure 2A) and displayed enhancement of hyperoxia-induced morphological alterations, including inflammatory infiltrates, edema, thickened alveolar walls and vascular congestion (Figure 2B). In addition, we examined the severity of lung injury and inflammation in mice exposed to hyperoxia. After 72 h of oxygen exposure, total cell count (Figure 2C), protein level (Figure 2D) and lactate dehydrogenase (LDH) activity (Figure 2E) in BALF were enhanced in CLU^−/−^ mice compared with those in WT mice. The level of 8-OH-dG, a biomarker of oxidative DNA damage caused by ROS, was significantly increased in the BALF of CLU^−/−^ mice exposed to hyperoxia for 72 h compared with levels in WT mice (Figure 2F).

Next, we examined the effect of CLU on cell death in a hyperoxia-induced lung injury mouse model using either WT or CLU^−/−^ mice. Compared to that in WT mice, lung alveolar cell apoptosis was enhanced in CLU^−/−^ mice, as determined by TUNEL staining (green) (Figure 2G). Caspase-3/7, caspase-8 and caspase-9 activities were also found to be elevated in CLU^−/−^ mouse lung after hyperoxia exposure (Figure 2H). The Fas/FasL pathway has previously been demonstrated to contribute to lung epithelial cell apoptosis in HALI [19,20]. Therefore, we examined the effect of CLU on the regulation of Fas expression. After exposure to oxygen for 72-h, CLU^−/−^ mice had significantly more enhancement in Fas mRNA expression than did WT mice (Figure 2I).

To assess whether CLU deficiency affects inflammatory response in the lungs during hyperoxia exposure, inflammatory cytokine levels were measured by real-time PCR and ELISA. Both IL-6 and TNF-α mRNA expression levels in CLU^−/−^ mouse lung were significantly higher than those in WT mouse lung following a 72 h hyperoxia exposure (Figure 2J). Furthermore, the protein levels of CCL2/MCP-1, IL-1β and CCL17/TARC were significantly increased in BALF from CLU^−/−^ mice compared to those in BALF from WT mice in the hyperoxia group (Figure 2K). These data indicate a relationship between the loss of airway CLU and significant inflammatory lung injury in mice subjected to prolonged hyperoxia exposure.

### 3.3. CLU Deficiency Increases Hyperoxia-Induced Apoptosis in Human Airway Epithelial Cells

**Figure 3:**
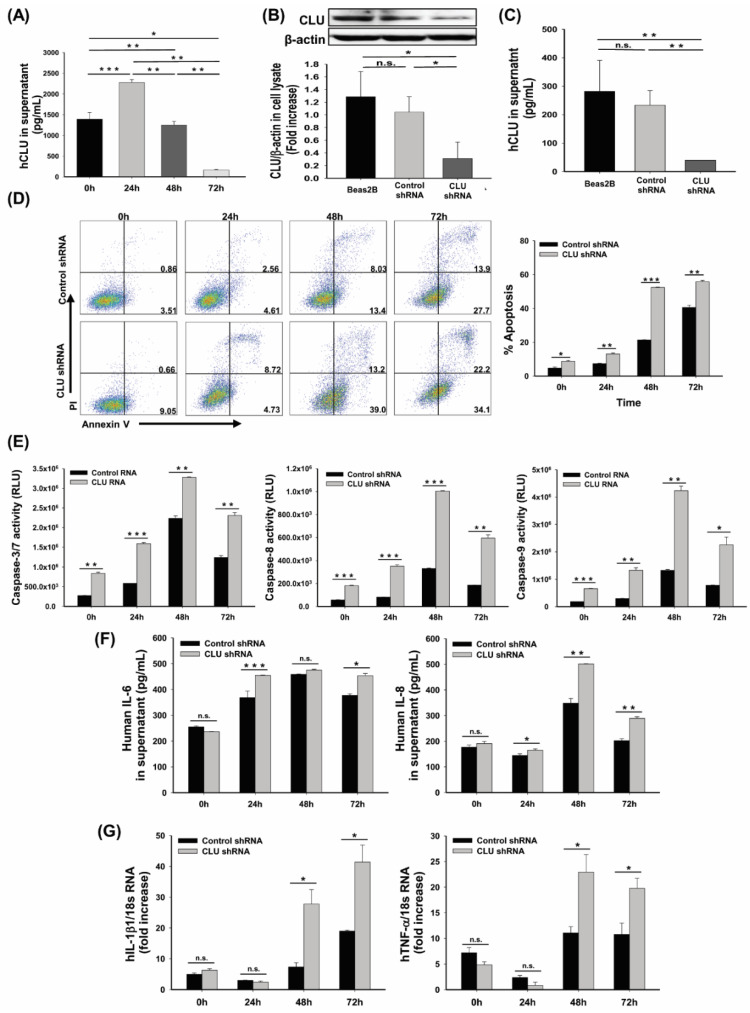
Hyperoxia induces apoptosis and inflammation in human airway epithelial cells. Beas-2B cells were cultured in 95% O_2_ and 5% CO_2_, for up to 72 h. (**A**) Clusterin (CLU) levels were measured in the supernatants by ELISA. Beas-2B cells were transfected with lentiviral CLU shRNA or control shRNA and (**B**) Western blotting and (**C**) ELISA showed the efficacy of shRNA knockdown of CLU in transformed Beas-2B cells. (**D**) Cell apoptosis was measured by annexin V/PI staining. Annexin V^+^PI^−^ and annexin V^+^PI^+^ cells were considered apoptotic cells. (**E**) Caspase-3/7, -8 and -9 activities were determined by Caspase-Glo^®^ 3/7 assay. (**F**) IL-6 and IL-8 levels were measured in the supernatant by ELISA. (**G**) The mRNA levels of IL-1β and TNF-α were detected by real-time PCR. Values represent the mean ± SEMs of three independent experiments. n.s. = not significant; * *p* < 0.05, ** *p* < 0.01, *** *p* < 0.001. RLU = relative luminescence units.

The airway epithelium contributes significantly to lung immunity and physiology after stimulation by oxidative stress in several ways [5,21]. Oxidative stress caused by cell apoptosis is mediated primarily by ROS. The release of superoxide anion O^−^ and hydrogen peroxide (H_2_O_2_), which are involved in the pathophysiological changes of these diseases, into the alveolar space triggers airway epithelium apoptosis [22]. CLU reportedly protects cells from a variety of stresses [9]; therefore, we tested whether the absence of CLU could cause oxidative stress-induced airway epithelial cell apoptosis. CLU protein levels were shown to increase in response to hyperoxia in human airway epithelial cells, Beas-2B and peaked at 24 h (Figure 3A). To examine the effects of reduced CLU expression on airway epithelial cell death, shRNA-mediated CLU-deficient (CLU shRNA) and control (mock-transduced; Control shRNA) airway epithelial cells were exposed to 95% O_2_ and 5% CO_2_ (Figure 3B,C). Apoptosis was quantified by flow cytometry using PI/Annexin V and caspase activity. Hyperoxia-exposed cells exhibited gradually increased early (Annexin V^+^PI^−^) and late apoptosis (Annexin V^+^PI^+^) rate after 24, 48, or 72 h of culture and CLU-deficient cells showed a higher apoptosis rate (Figure 3D). In addition, the activities of caspase-3/7, -8 and -9 increased and peak at 48 h after exposure to hyperoxia. Furthermore, the caspase activities were increased in CLU shRNA cells compared with levels in control shRNA cells (Figure 3E). Moreover, inflammatory cytokine mRNA expression and concentration in the supernatant of hyperoxia-exposed CLU shRNA cells were significantly higher than those in the control shRNA cells (Figure 3F,G).

### 3.4. CLU Regulates Mitochondrial Potential and Apoptosis in Hyperoxia-Exposed Mice and Human Airway Epithelial Cells

**Figure 4:**
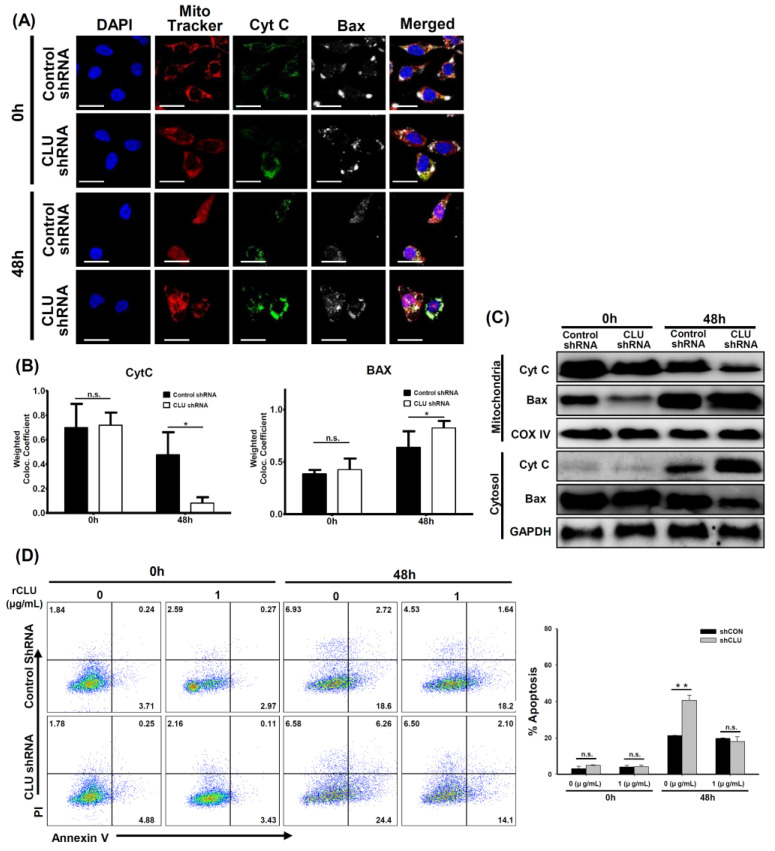
Clusterin (CLU) regulates the levels of Bax and cytochrome c and apoptosis. Wild-type (WT) and CLU-deficient (CLU^−/−^) mice were exposed to >95% O_2_ for 48 h. (**A**) Immunofluorescence experiments were conducted. Double staining against MitoTracker (red)/cytochrome c (Cyt C) (green) and MitoTracker (red)/Bax (white) is shown. (**B**) Mean intensity of co-localization with MitoTracker (Red) was calculated using the ZEN software. (**C**) Protein expression of Bax and Cyt C in both cytosolic and mitochondrial fractions was observed by Western blotting. (**D**) Cells were cultured in 95% O_2_ and 5% CO_2_ with recombinant CLU (rCLU) for up to 48 h. Cellular apoptosis was assayed by annexin V and PI counterstaining and analyzed with flow cytometry. Scale bars in (**A**); 20 μm, n.s. = not significant; * *p* < 0.05, ** *p* < 0.01.

Hyperoxia exposure resulted in the activation of Bax at the mitochondrial membrane, cytochrome c release and cell death [23]. Release of mitochondrial cytochrome c, which induces cleavage of caspase-9, into the cytosol is a key event in hyperoxia-induced lung injury [24,25]. We further evaluated cytochrome c release and Bax translocation in both the cytosol and mitochondria. In the 0 h control cells, cytochrome c (green) co-localized with the MitoTracker (red), but Bax (white) was found to be localized throughout the cytosol (Figure 4A). However, mitochondrial localization of cytochrome c decreased with time, as the mitochondrial membrane potential was reduced after hyperoxia exposure (Figure 4A,B). In addition, Western blotting demonstrated an increase in cytosolic cytochrome c and mitochondrial Bax compared with levels in the 0 h control cells (Figure 4C).

To determine the effects of CLU recovery on hyperoxia-induced cell death, CLU-deficient cells were cultured with 1 µg/mL rCLU during hyperoxia. This reduced cell death progression of hyperoxia-induced cells when compared with untreated-controls (Figure 4D). These data demonstrated that CLU may also have a role in protecting cells from hyperoxia-induced apoptosis.

### 3.5. Analysis of DEGs and Pathways Based on Exposure to Hyperoxia

**Figure 5:**
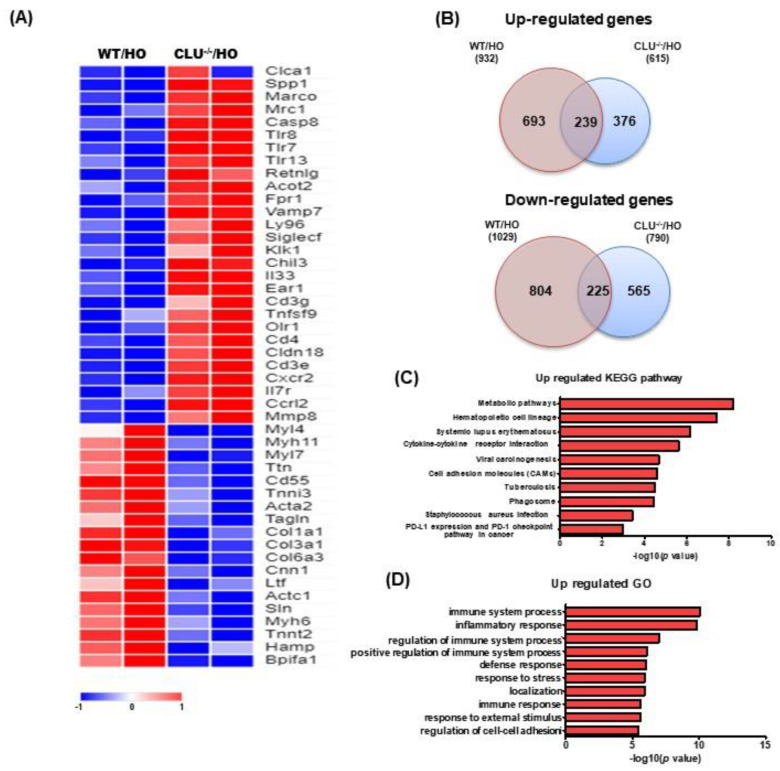
Microarray and pathway analysis of RNA from hyperoxia-induced acute lung injury. (**A**) Heat map representing differences in the expression of genes, as indicated. (WT/HO *n* = 2, CLU^−/−^/HO *n* = 2 animals per group) (**B**) Diagrams showing the number of upregulated and downregulated genes in WT and CLU^−/−^ mice under hyperoxic conditions. (**C**) Kyoto Encyclopedia of Genes (KEGG) pathway and (**D**) Gene Ontology (GO) analyses of significant differentially expressed genes were conducted. The bars represent -log(*p*-value); the number of statistically significant genes is indicated in each category.

Next, we performed microarray analysis to gain insight into the genome-wide transcriptional changes accompanying CLU deficiency in lungs from room air control or hyperoxia-exposed mice (adjusted *p*-value < 0.05, fold change > 2). Heatmap showed DEGs between CLU^−/−^ and WT mice under hyperoxic conditions (Figure 5A). We also compared the number of genes that showed changes in expression in hyperoxia-exposed CLU^−/−^ (CLU^−/−^/HO) mice to that in hyperoxia-exposed WT (WT/HO) mice. We found that 693 and 376 genes were upregulated in the CLU^−/−^ and WT groups, respectively, with 239 genes being mutually upregulated in both groups when compared with room air control mice (Figure 5B). Next, we elucidated how the DEGs affect biological pathways in CLU^−/−^ mice compared with WT mice under hyperoxia using the KEGG and Gene Ontology (GO) databases. Upregulated pathways associated with CLU changes in hyperoxia-induced acute lung injury included metabolic pathway, hematopoietic cell lineage, systemic lupus erythematosus, cytokine-cytokine receptor interaction, viral carcinogenesis, cell adhesion molecules (CAMs), tuberculosis, phagosome, *Staphylococcus aureus* infection and PD-L1 expression and PD-1 checkpoint (Figure 5C and Table 1). The top 10 pathways based on the interaction between CLU gene and hyperoxia were related to immune system, inflammatory response, regulation of immune system, positive regulation of immune system, defense response, response to stress, localization, immune response, response to external stimulus and regulation of cell-cell adhesion (Figure 5D).

## 4. Discussion

In this study, we observed increased CLU expression in the lungs of mice and humans and in cultured epithelial cells subjected to prolonged oxygen exposure. Moreover, we demonstrated a phenotype of high concentration oxygen-mediated toxicity in CLU^−/−^ mice, including reinforced lung injury, increased inflammatory response and decreased survival rate compared with observations in WT mice.

CLU is a multifunctional glycoprotein that is involved in various biological processes, such as cell survival, differentiation, migration, proliferation [8]. CLU has two isotypes, namely the secretory/cytoplasmic form (sCLU) and the nuclear form (nCLU) [26]. sCLU is widely involved in the pro-survival mechanism of various cells and mouse models. Indeed, several recent studies have suggested that CLU interacts with several protein complexes known to be involved in apoptosis pathways [8,26,27]. A potential mechanism by which CLU exerts its anti-apoptotic effects has been suggested. CLU is upregulated by apoptotic triggers and then it inhibits apoptosis by associating with the pro-apoptotic Bcl-2-associated protein Bax in the cytoplasm to alter the conformation of the Ku70/Bax complex [27]. A previous study demonstrated that most of the conformation-altered Bax was co-localized with CLU following camptothecin stimulation and that elevated CLU interacted with conformation-altered Bax, thereby inhibiting activation of the release of cytochrome c from mitochondria [28]. In addition, it has been indicated that stimulus-induced CLU expression prevented the translocation of activated Bax to mitochondria through the activation of NF-κB signaling and phosphorylation of ERK and AKT in some cancer cells [12,26,27]. In this study, elevated levels of CLU were observed in the hyperoxia-induced acute lung injury model (Figure 1) and human airway epithelial cells (Figure 3A). Moreover, CLU deficiency deteriorated all the parameters of lung damage, such as mortality (Figure 2A), cell death (Figure 2G,H and Figure 3D,E) and oxidative stress (Figure 2F). Further, our findings identified CLU to also regulate both the translocation of Bax and release of cytochrome c (Figure 4A–C) and to decrease apoptosis rate (Figure 4D). Based on these results and the literature, one possible mechanism underlying the protective function of CLU in hyperoxia-induced injury would involve regulation of CLU production by Bax-mediated apoptosis. Thus, a low concentration of CLU would diminish the stability of the Ku70/Bax complex in the cytoplasm, facilitating Bax translocation to mitochondria to induce cytochrome c release and, thus, activating a caspase cascade and the resultant apoptosis.

In a steady state, clearing of apoptotic cells can maintain tissue homeostasis and prevent the initiation of immune responses [29]. In contrast, dysregulation of clearance of dying cells can stimulate the release of inflammatory mediators that induce initiation of the innate and/or adaptive immune response [30]. sCLU is ubiquitously expressed in most mammalian tissues and body fluids, such as blood plasma, urine, semen, breast milk and cerebrospinal fluid [8]. Previously, it was shown that sCLU acts as a chaperone molecule, similar to a small heat shock protein, allowing the clearance of cellular debris and misfolded proteins [8,31]. This chaperone activity may be one way of protecting tissues from many injuries [32]. A recent study demonstrated that CLU-deficient mice were more sensitive to and showed a more intense immune response to injected apoptotic cells. Importantly, CLU promotes the clearance of late apoptotic cells by binding to histones on the surface of late apoptotic cells and this binding of CLU to histones is independent of cell type or stress [32]. After hyperoxia exposure, the levels of inflammatory cytokine expression were increased in both CLU^−/−^ mice and CLU shRNA cells compared with levels in WT and controls (Figure 2J,K and Figure 3F,G). In addition, the results of GO analysis indicated that the DEGs associated with the CLU^−/−^/HO group were significantly enriched in immune system process, among others (Figure 5). Our results indicate that the absence of CLU may have contributed to a defect in apoptotic cell clearance and/or an excess of apoptotic cells, causing activation of the immune response.

The administration of high levels of oxygen to animal results in a significant pulmonary toxicity, characterized by pronounced inflammatory responses with influx of inflammatory cells, increased pulmonary permeability and injury/death of pulmonary cells, including epithelial cells, endothelial cells, neutrophils and macrophages [1,3,4,33]. These inflammatory cells in the alveolar space exacerbate the lung injury and may act as a potent source of inflammatory cytokines, including TNF-α, MIP-2, IL-1β, IL-6, MCP-1 and IL-8, which are crucial mediators in the early stages of inflammatory responses [29,34]. In support of this notion, we identified that hyperoxia exposure enhanced the expression of inflammatory cytokines (IL-6, IL-8, TNF-α, MCP-1 and IL-1β) in the CLU-deficient group compared with that in the controls (Figure 2J,K and Figure 3F,G). IL-6 and IL-1β reportedly increased in the lungs of adult and newborn mice, accompanied by lethality [30] and hyperoxia also reportedly resulted in an increase in IL-8 level in mouse BALF and human tracheal aspirate and could also act as an important inducer of the influx of neutrophils [35]. Necrotic cells (cellular RNA) stimulate the synthesis of cytoprotective sCLU and the chemokine MCP-1 via TLR3 in rat vascular smooth muscle cells. Treatment with MCP-1 induces monocyte differentiation into macrophages, which are involved in phagocytosis and the immune response [29]. Moreover, we found enhanced levels of chemokine expression (Figure 2K) and infiltration of inflammatory cells (Figure 2B,C) in CLU^−/−^ mice compared with WT mice exposed to hyperoxia. It has been demonstrated that elevated chemokine expressions such as CCL2 and CXCL1 led to inflammatory cell infiltration into the lungs during hypeoxia exposure [3]. Thus, it is possible that the exacerbated lung injury in CLU^−/−^ mice might be induced by altered cell death cascade and persistent inflammatory response in this study.

Prolonged exposure to oxygen can lead to persistent damage and death of lung epithelial cells; it is associated with abnormal lung tissue repair and inflammation after hyperoxic lung injury [36]. Hyperoxia causes acute respiratory distress syndrome in adults and leads to a pulmonary phenotype suggestive of BPD in neonates [37]. Thus, HALI is one of the major causes of morbidity and mortality in both adult and neonatal populations [37,38]. BPD is a commonly prevalent lung disease in preterm neonates and can be affected by several factors, including inflammation and exposure to oxygen [36]. Our findings identify CLU as a critical negative regulator of hyperoxic epithelial cell death and inflammation; thus, CLU could be a therapeutic target and may improve clinical implications for HALI. Furthermore, understanding the regulation of immature lung injury and the mechanisms of protection against apoptosis and inflammation during hyperoxia might lead to identification of other specific signaling pathways. Hence, further investigations into the role of CLU in the hyperoxia-induced BPD models and into genetic variation, inflammation and other factors concerning the pathogenesis of BPD mechanism are necessary.

In summary, our study identified CLU as a potential factor protecting alveolar cells from apoptosis and inflammation during hyperoxia-induced acute lung injury in vivo and hyperoxia stress in vitro. The major findings of this study are as follows. First, both lung and Beas-2B cells showed increased CLU expression during hyperoxia. Second, mortality was markedly increased in CLU^−/−^ mice compared with that in WT mice under hyperoxia and the enhanced lethality was associated with the acceleration of acute lung injury. Moreover, CLU deficiency exacerbated hyperoxia-induced cell apoptosis, whereas CLU restoration alleviated the cell damage. Lastly, CLU provided protection against hyperoxia-induced mitochondrial membrane disruption as well as prevented Bax translocation, cytochrome *c* release and caspase activation. In addition, microarray analysis revealed that metabolism, hematopoietic cell lineage, immune function and cytokine signaling pathways were upregulated in CLU^−/−^ mice.

Taken together, our findings suggest that during hyperoxia induced-acute lung injury, CLU is a negative regulator of pulmonary permeability change, inflammatory cell accumulation in alveoli and cell death cascade.

## Figures and Tables

**Table 1:** Detailed analysis of Kyoto Encyclopedia of Genes and Genomes (KEGG) pathways.

Term	*p*-Value	Number of DEGs	DEGs
Metabolic pathways	6.2063 × 10^−9^	28	Cth, Prps2, Alas2, Aldoc, Alox5, Bst1, Bpgm, Cat, Cyp3a13, Cyp51, Hnmt, Gcnt2, Lss, Acot2, Rdh11, Pla2g1b, Sephs2, Man1c1, Nt5e, Papss2, Atp6v0d2, Uprt, Gatc, Sgpp2, Coq5, Mocs1, Lipf, Chia1
Hematopoietic cell lineage	3.7106 × 10^−8^	9	Ms4a1, Cd33, Cd3e, Cd3g, Cd4, Csf2ra, Gypa, Il7r, Gp9
Systemic lupus erythematosus	6.7975 × 10^−7^	9	C6, Fcgr4, Hist1h4c, Hist1h4d, Hist1h4i, Hist1h4n, Hist1h2bg, Hist4h4, Hist1h4h
Cytokine-cytokine receptor interaction	2.3806 × 10^−6^	11	LOC100041504, Gm13304, Gm1987, Bmp4, Cd4, Cxcr2, Csf2ra, Il1rn, Il7r, Tnfsf9, Il33
Viral carcinogenesis	2.1249 × 10^−5^	9	Casp8, Atp6v0d2, Hist1h4c, Hist1h4d, Hist1h4i, Hist1h4n, Hist1h2bg, Hist4h4, Hist1h4h
Cell adhesion molecules (CAMs)	2.5415 × 10^−5^	8	Cd4, Itgb2, Selp, Selplg, Siglec1, Cd226, Cldn18, Cd274
Tuberculosis	3.2532 × 10^−5^	8	Casp8, Itgax, Itgb2, Mrc1, Atp6v0d2, Fcgr4, Clec4e, Clec7a
Phagosome	3.4842 × 10^−5^	8	Olr1, Cybb, Itgb2, Marco, Mrc1, Atp6v0d2, Fcgr4, Clec7a
*Staphylococcus aureus* infection	0.0003783	6	Fpr2, Fpr1, Itgb2, Selp, Selplg, Fcgr4
Alcoholism	0.00050774	7	Hist1h4c, Hist1h4d, Hist1h4i, Hist1h4n, Hist1h2bg, Hist4h4, Hist1h4h
PD-L1 expression and PD-1 checkpoint pathway in cancer	0.00102848	5	Cd3e, Cd3g, Cd4, Ptpn6, Cd274

DEGs, differentially expressed genes.

## Data Availability

Not applicable.

## References

[1] Sohn M.H., Kang M., Matsuura H., Bhandari V., Chen N., Lee C.G., Elias J.A. (2010). The chitinase-like proteins breast regression protein-39 and YKL-40 regulate hyperoxia-induced acute lung injury. Am. J. Respir. Crit. Care Med..

[2] Laube M., Amann E., Uhlig U., Yang Y., Fuchs H.W., Zemlin M., Mercier J.-C., Maier R.F., Hummler H.D., Uhlig S. (2017). Inflammatory Mediators in Tracheal Aspirates of Preterm Infants Participating in a Randomized Trial of Inhaled Nitric Oxide. PLoS ONE.

[3] Mizushina Y., Shirasuna K., Usui F., Karasawa T., Kawashima A., Kimura H., Kobayashi M., Komada T., Inoue Y., Mato N. (2015). NLRP3 Protein Deficiency Exacerbates Hyperoxia-induced Lethality through Stat3 Protein Signaling Independent of Interleukin-1β. J. Biol. Chem..

[4] Bhandari V., Choo-Wing R., Homer R.J., Elias J.A. (2007). Increased Hyperoxia-Induced Mortality and Acute Lung Injury in IL-13 Null Mice. J. Immunol..

[5] Budinger G.R.S., Mutlu G.M., Urich D., Soberanes S., Buccellato L.J., Hawkins K., Chiarella S.E., Radigan K.A., Eisenbart J., Agrawal H. (2011). Epithelial Cell Death Is an Important Contributor to Oxidant-mediated Acute Lung Injury. Am. J. Respir. Crit. Care Med..

[6] Qin S., Chen M., Ji H., Liu G., Mei H., Li K., Chen T. (2018). miR-21-5p regulates type II alveolar epithelial cell apoptosis in hyperoxic acute lung injury. Mol. Med. Rep..

[7] Pereira D.M.L., Mekary R.A., Rodrigues K.C.D.C., Anaruma C.P., Ropelle E.R., Da Silva A.S.R., Cintra D.E., Pauli J.R., De Moura L.P. (2018). Protective molecular mechanisms of clusterin against apoptosis in cardiomyocytes. Heart Fail. Rev..

[8] Rohne P., Prochnow H., Koch-Brandt C. (2016). The CLU-files: Disentanglement of a mystery. Biomol. Concepts.

[9] Hong G.H., Kwon H.-S., Moon K.-A., Park S.Y., Park S., Lee K.Y., Ha E.H., Kim T.-B., Moon H.-B., Lee H.K. (2016). Clusterin Modulates Allergic Airway Inflammation by Attenuating CCL20-Mediated Dendritic Cell Recruitment. J. Immunol..

[10] Bae C.H., Na H.G., Choi Y.S., Song S., Kim Y. (2018). Clusterin Induces MUC5AC Expression via Activation of NF-κB in Human Airway Epithelial. Cells Clin. Exp. Otorhinolaryngol..

[11] Trougakos I.P., So A., Jansen B., Gleave M.E., Gonos E.S. (2004). Silencing expression of the clusterin/apolipoprotein j gene in human cancer cells using small interfering RNA induces spontaneous apoptosis, reduced growth ability, and cell sensitization to genotoxic and oxidative stress. Cancer Res..

[12] Jun H., Kim D., Lee S., Lee H.S., Seo J.H., Kim J.H., Yu Y.S., Min B.H., Kim K. (2011). Clusterin protects H9c2 cardiomyocytes from oxidative stress-induced apoptosis via Akt/GSK-3β signaling pathway. Exp. Mol. Med..

[13] Habiel D.M., Camelo A., Espindola M., Burwell T., Hanna R., Miranda E., Carruthers A., Bell M., Coelho A.L., Liu H. (2017). Divergent roles for Clusterin in Lung Injury and Repair. Sci. Rep..

[14] Takase O., Minto A.W., Puri T.S., Cunningham P.N., Jacob A., Hayashi M., Quigg R.J. (2008). Inhibition of NF-kappaB-dependent Bcl-xL expression by clusterin promotes albumin-induced tubular cell apoptosis. Kidney Int..

[15] Carnevali S., Luppi F., D’Arca D., Caporali A., Ruggieri M.P., Vettori M.V., Caglieri A., Astancolle S., Panico F., Davalli P. (2006). Clusterin Decreases Oxidative Stress in Lung Fibroblasts Exposed to Cigarette Smoke. Am. J. Respir. Crit. Care Med..

[16] Hong J.Y., Na Kim M., Kwak E.J., Kim E.G., Kim K.W., Sohn M.H. (2019). Role of Clusterin in Protection Against Hyperoxic Lung Injury in Mice. Mech. Lung Inj. Repair.

[17] Hong J.Y., Kim M., Sol I.S., Kim K.W., Lee C.-M., Elias J.A., Sohn M.H., Lee C.-M., Lee C.G. (2018). Chitotriosidase inhibits allergic asthmatic airways via regulation of TGF-β expression and Foxp3+ Treg cells. Allergy.

[18] Kim M.J., Hong J.Y., Lee K.E., Kim K.W., Sohn M.H., Kim K.-E. (2013). Effect of Cholesterol Depletion on Interleukin-8 Production in Human Respiratory Epithelial Cells. Allergy Asthma Immunol. Res..

[19] De Paepe M.E., Mao Q., Chao Y., Powell J.L., Rubin L.P., Sharma S. (2005). Hyperoxia-induced apoptosis and Fas/FasL expression in lung epithelial cells. AJP-Lung Cell. Mol. Physiol..

[20] Albertine K.H., Soulier M.F., Wang Z., Ishizaka A., Hashimoto S., Zimmerman G.A., Matthay M.A., Ware L.B. (2002). Fas and Fas Ligand Are Up-Regulated in Pulmonary Edema Fluid and Lung Tissue of Patients with Acute Lung Injury and the Acute Respiratory Distress Syndrome. Am. J. Pathol..

[21] Lee K.E., Jee H.M., Hong J.Y., Na Kim M., Oh M.S., Kim Y.S., Kim K.W., Kim K.E., Sohn M.H. (2018). German Cockroach Extract Induces Matrix Metalloproteinase-1 Expression, Leading to Tight Junction Disruption in Human Airway Epithelial Cells. Yonsei Med. J..

[22] Wang Y., Lee C.G.L. (2008). MicroRNA and cancer-focus on apoptosis. J. Cell. Mol. Med..

[23] Buccellato L.J., Tso M., Akinci O.I., Chandel N.S., Budinger G.R.S. (2004). Reactive Oxygen Species Are Required for Hyperoxia-induced Bax Activation and Cell Death in Alveolar Epithelial Cells. J. Biol. Chem..

[24] Chowdhury S.R., Sengupta S., Biswas S., Sinha T.K., Sen R., Basak R.K., Adhikari B., Bhattacharyya A. (2014). Bacterial fucose-rich polysaccharide stabilizes MAPK-mediated Nrf2/Keap1 signaling by directly scavenging reactive oxygen species during hydrogen peroxide-induced apoptosis of human lung fibroblast cells. PLoS ONE.

[25] Pagano A., Donati Y., Métrailler I., Argiroffo C.B. (2004). Mitochondrial cytochrome c release is a key event in hyperoxia-induced lung injury: Protection by cyclosporin A. Am. J. Physiol. Cell. Mol. Physiol..

[26] Koltai T. (2014). Clusterin: A key player in cancer chemoresistance and its inhibition. OncoTargets Ther..

[27] Zhang H., Kim J.K., Edwards C.A., Xu Z., Taichman R., Wang C.-Y. (2005). Clusterin inhibits apoptosis by interacting with activated Bax. Nat. Cell Biol..

[28] Trougakos I.P., Lourda M., Antonelou M.H., Kletsas D., Gorgoulis V.G., Papassideri I.S., Zou Y., Margaritis L.H., Boothman D.A., Gonos E.S. (2009). Intracellular Clusterin Inhibits Mitochondrial Apoptosis by Suppressing p53-Activating Stress Signals and Stabilizing the Cytosolic Ku70-Bax Protein Complex. Clin. Cancer Res..

[29] Klock G., Baiersdörfer M., Koch-Brandt C. (2009). Chapter 7: Cell protective functions of secretory Clusterin (sCLU). Adv. Cancer Res..

[30] Patel V.A., Longacre A., Hsiao K., Fan H., Meng F., Mitchell J.E., Rauch J., Ucker D.S., Levine J.S. (2006). Apoptotic cells, at all stages of the death process, trigger characteristic signaling events that are divergent from and dominant over those triggered by necrotic cells: Implications for the delayed clearance model of autoimmunity. J. Biol. Chem..

[31] Stewart E.M., Aquilina J.A., Easterbrook-Smith S.B., Murphy-Durland D., Jacobsen C., Moestrup S.K., Wilson M.R. (2007). Effects of Glycosylation on the Structure and Function of the Extracellular Chaperone Clusterin. Biochemistry.

[32] Cunin P., Beauvillain C., Miot C., Augusto J.-F., Preisser L., Blanchard S., Pignon P., Scotet M., Garo E., Fremaux I. (2016). Clusterin facilitates apoptotic cell clearance and prevents apoptotic cell-induced autoimmune responses. Cell Death Dis..

[33] Bhandari V., Choo-Wing R., Lee C.G., Zhu Z., Nedrelow J.H., Chupp G.L., Zhang X., Matthay M.A., Ware L.B., Homer R.J. (2006). Hyperoxia causes angiopoietin 2–mediated acute lung injury and necrotic cell death. Nat. Med..

[34] Bhandari V., Elias J.A. (2006). Cytokines in tolerance to hyperoxia-induced injury in the developing and adult lung. Free Radic. Biol. Med..

[35] D’Angio C.T., Lomonaco M.B., Chaudhry S.A., Paxhia A., Ryan R.M. (1999). Discordant pulmonary proinflammatory cytokine expression during acute hyperoxia in the newborn rabbit. Exp. Lung Res..

[36] Higgins R.D., Jobe A.H., Koso-Thomas M., Bancalari E., Viscardi R.M., Hartert T.V., Ryan R.M., Kallapur S.G., Steinhorn R.H., Konduri G.G. (2018). Bronchopulmonary Dysplasia: Executive Summary of a Workshop. J. Pediatr..

[37] Buczynski B.W., Maduekwe E.T., O’Reilly M.A. (2013). The Role of Hyperoxia in the Pathogenesis of Experimental BPD. Semin. Perinatol..

[38] Wang J., Dong W. (2018). Oxidative stress and bronchopulmonary dysplasia. Gene.

